# Membranoproliferative glomerulonephritis presenting as arthropathy and cardiac valvulopathy in hypocomplementemic urticarial vasculitis: a case report

**DOI:** 10.1186/1752-1947-8-352

**Published:** 2014-10-22

**Authors:** Chuiyoung Park, Seung Won Choi, Misung Kim, Jongha Park, Jong Soo Lee, Hyun Chul Chung

**Affiliations:** 1 Department of Internal Medicine, Ulsan University Hospital, 290-3 Jeonha-dong, Dong-gu, Ulsan 682-714, Republic of Korea; 2 Department of Pathology, Ulsan University Hospital, 290-3 Jeonha-dong, Dong-gu, Ulsan 682-714, Republic of Korea

**Keywords:** Hypocomplementemic urticarial vasculitis syndrome, Arthropathy, Valvulopathy, Membranoproliferative glomerulonephritis

## Abstract

**Introduction:**

Hypocomplementemic urticarial vasculitis syndrome is a rare disorder characterized by chronic urticarial vasculitis, arthralgia, arthritis, and hypocomplementemia. Previously, only six patients with concomitant hypocomplementemic urticarial vasculitis syndrome, Jaccoud’s arthropathy, and valvular heart disease have been reported.

**Case presentation:**

A 30-year-old Korean man presented with hypocomplementemic urticarial vasculitis syndrome. In addition to urticarial cutaneous lesions, he experienced polyarthralgia and arthritis that resulted in progressive deformity of the joints of both hands, cardiac valvulopathy with mitral, tricuspid, and aortic regurgitation, and intermittent neck swelling with laryngeal edema. He also developed nephritis with azotemia. His renal biopsy results revealed membranoproliferative glomerulonephritis, type I. He showed a partial response to a combination therapy of steroid, cyclophosphamide, and mycophenolate mofetil.

**Conclusions:**

We describe, to the best of our knowledge, the first case of glomerulonephritis presenting a arthropathy and cardiac valvulopathy in hypocomplementemic urticarial vasculitis syndrome. A combination of corticosteroids, cyclophosphamide, and mycophenolate mofetil appear to be a safe and effective treatment for nephropathy, however are less effective for cutaneous vasculitis, cardiac valvulopathy, and arthropathy.

## Introduction

Hypocomplementemic urticarial vasculitis syndrome (HUVS) is a rare disorder characterized by chronic urticarial vasculitis, arthralgia, arthritis, and activation of the classical complement pathway. Most patients have autoantibodies to C1q [1]. Previously, only six patients with concomitant HUVS, Jaccoud’s arthropathy, and valvular heart disease have been reported [2-5]. In this case, we describe a 30-year-old Korean man with this rare combination of symptoms, as well as azotemia secondary to membranoproliferative glomerulonephritis.

## Case presentation

**Figure 1:**
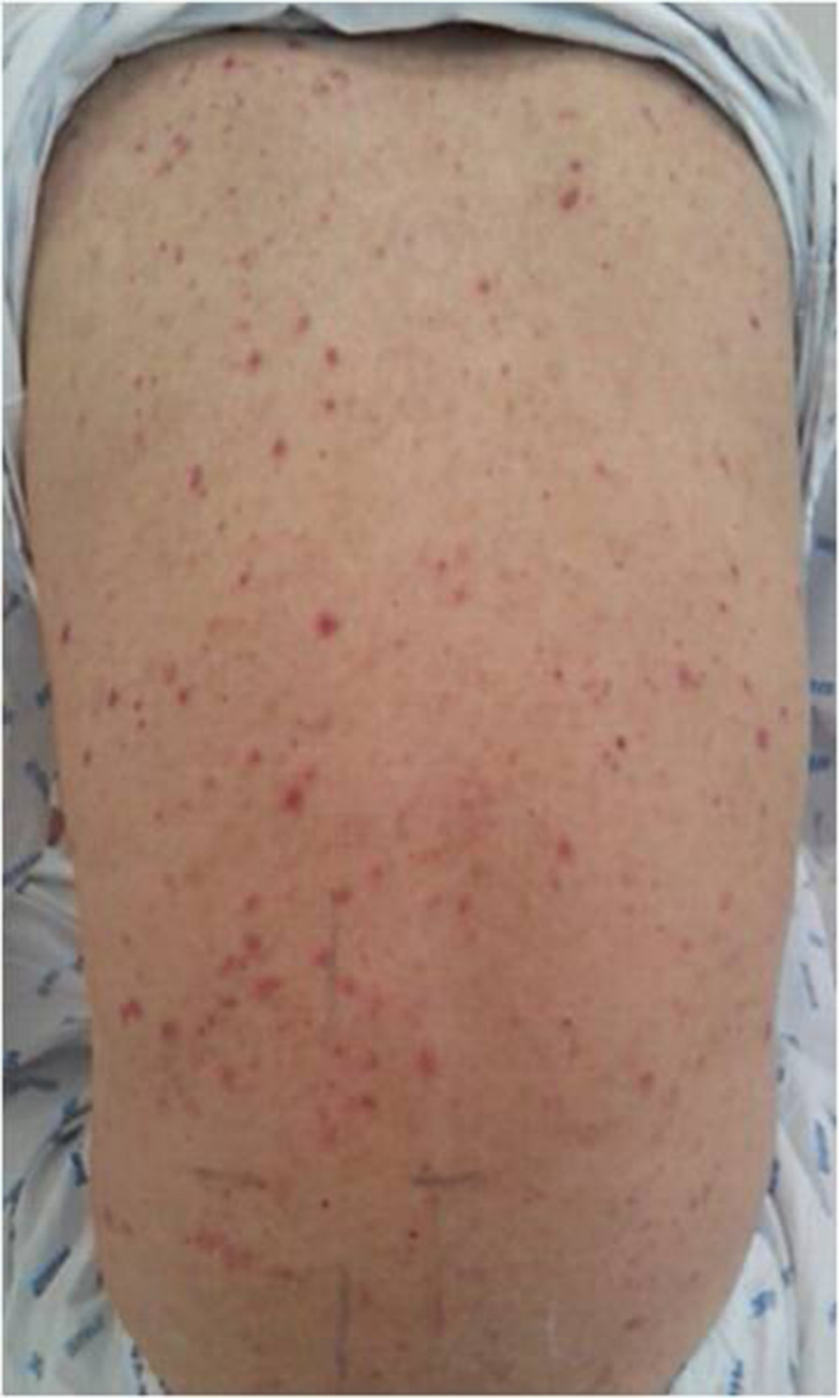
Urticarial rash and hyperpigmentation.

A 30-year-old Korean man presented to our hospital with recurrent urticarial lesions and joint pain for one year previous. The urticarial lesions resolved with hyperpigmentation or residual purpura. The arthralgias were intermittent and migratory, affecting the hands, knees, and ankles, and resolved spontaneously after 2 to 3 days. He did not smoke, denied any use of alcohol or herbal remedies, and had no family history of autoimmune disease. On examination diffusely scattered, variably sized erythematous pruritic papules were noted on his trunk and extremities. Laboratory test results revealed his levels of blood urea nitrogen (BUN) to be 12.3mg/dL, serum creatinine 1.09mg/dL, C-reactive protein 1.167mg/L, and rheumatoid factor (RF) <2.5IU/mL (normal range between 0 and 14IU/mL). He was found to be negative for anti-cyclic citrullinated peptide antibody (anti-CCP), antinuclear antibodies (ANA) titer 1:40, speckled type, and the HLA-B27 genotype was negative, and his erythrocyte sedimentation rate (ESR) was 21mm/h. No cartilage or bone destruction was detected on radiographs of his hands. He was diagnosed with rheumatoid factor-negative rheumatoid arthritis with skin vasculitis and was treated with prednisolone, hydroxychloroquine, methotrexate, and non-steroidal anti-inflammatory drugs.Two years after his initial visit, he was admitted to our hospital due to a worsening skin rash, right flank pain, and upper abdominal pain unresponsive to medication. His blood pressure was 148/80mm Hg, his heart rate was regular at 76 beats/min, and his body temperature was 36.3°C. He had diffuse urticarial lesions on his face, trunk, and upper arms (Figure 1). A skin biopsy of a new urticarial lesion revealed leukocytoclastic vasculitis. An abdominal computed tomography (CT) scan revealed multifocal low-attenuated lesions in both kidneys without other abnormalities. A laboratory workup showed his levels of total protein to be 5.7g/dL, albumin 3.3g/dL, BUN 21mg/dL, creatinine 1.38mg/dL, D-dimer 3.5μg/mL (normal range, 0 to 1μg/mL), fibrinogen 481mg/dL (normal range, 200 to 400mg/dL), lactate dehydrogenase 751IU/L (normal range 218 to 472IU/L), C-reactive protein 5.261mg/L, normal prothrombin time and activated partial thromboplastin time; antithrombin III 90% (normal range, 80 to 120%), normal protein C and S activities, and his ESR was 31mm/h.

**Figure 2:**
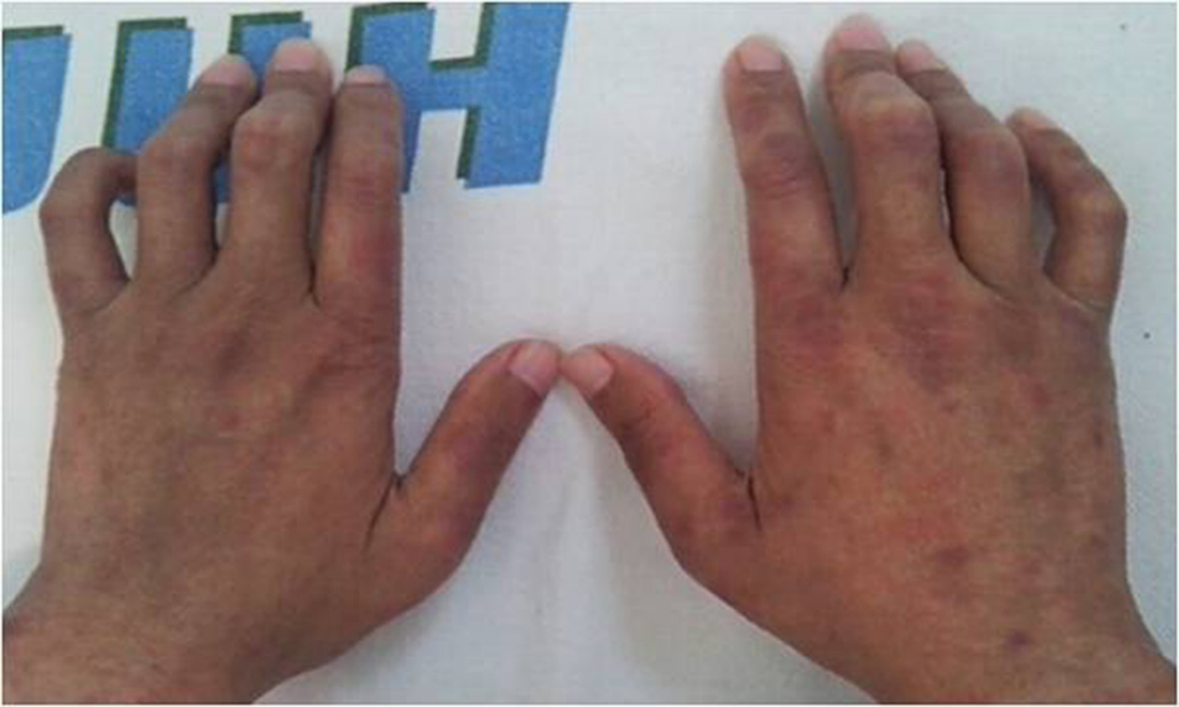
Swelling of the proximal interphalangeal joints with swan neck and flexion deformities of both hands.

**Figure 3:**
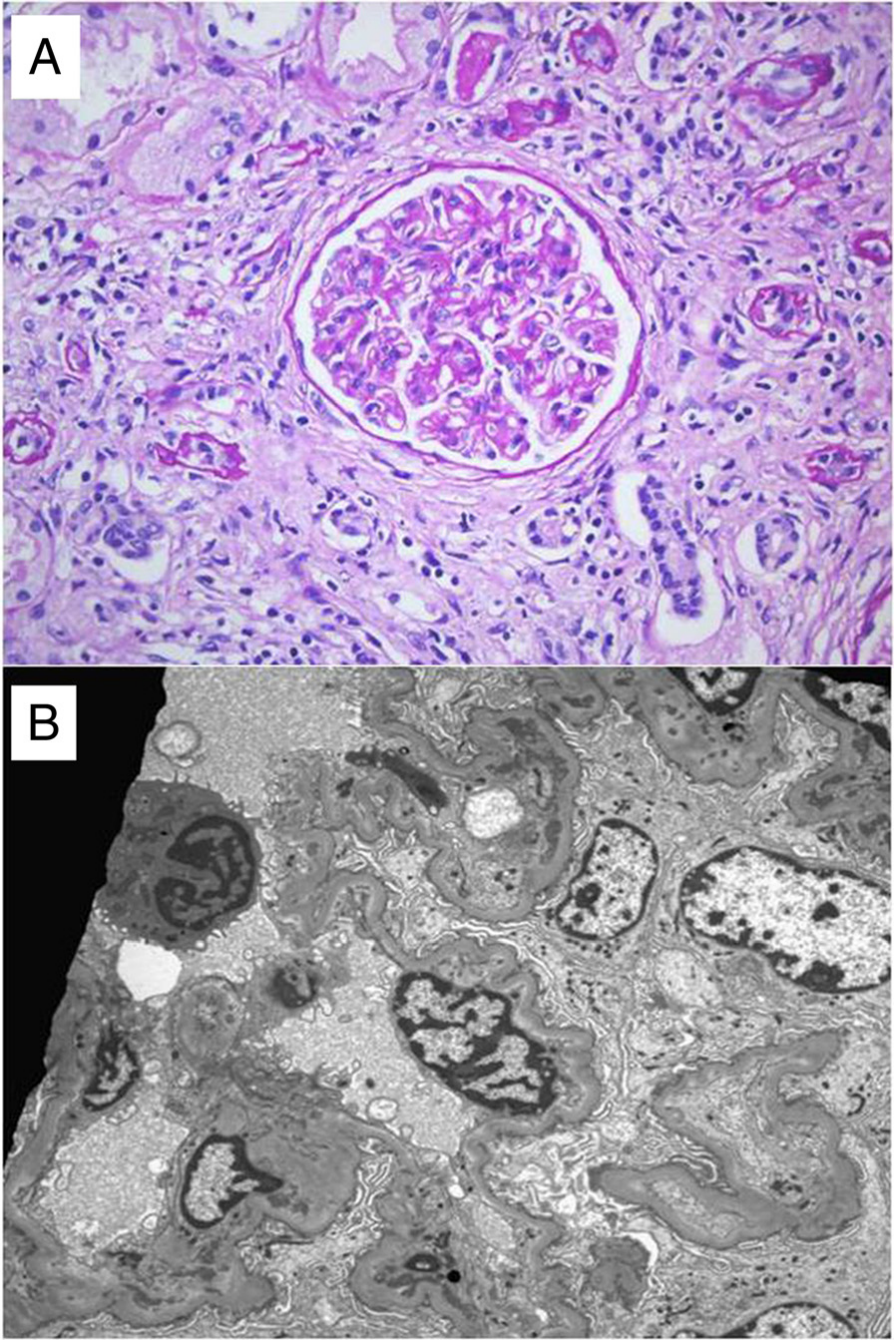
**Findings of kidney biopsy. (A)** The mesangium was expanded due to increased matrix without hypercellularity. The glomerular capillary walls were mildly thickened. Peridic acid-Schiff (PAS) stain; original magnification ×400. **(B)** Electron microscopy shows irregular electron dense deposits in the subendothelial and paramesangial space.

He was found to be negative for ANA, anti-double-stranded DNA antibody, anti-Smith antibody, anti-SS-A/SS-B, lupus anticoagulant, antistreptolysin O, and antineutrophil cytoplasmic antibody (ANCA). The concentrations of serum IgG, IgA, and IgM were normal. His C3 level was 12.2mg/dL (normal range 90 to 180mg/dL), C4 level <1.5mg/dL (normal range 10 to 40mg/dL), and CH50 level 4.2U/mL (normal range 23 to 46U/mL), and his cryoglobulin test result was negative. His serological test results for hepatitis B and C viruses were also negative. His urine analysis showed trace protein, red blood cell (RBC) count 0 to 1/high-power field (HPF), and a urine protein-creatinine ratio of 364.4mg/g. A chest radiograph showed no abnormalities. An echocardiogram showed moderate mitral and tricuspid regurgitation with moderate-to-severe aortic regurgitation. There was diastolic left ventricular dysfunction and normal left ventricular systolic function. An aortography, superior mesenteric, celiac, and bilateral renal arteriography revealed no abnormalities. High-dose oral prednisolone (1mg/kg) was administered for the treatment of active vasculitis. His abdominal pain and skin lesions subsequently improved.One year after continuous treatment with steroid and azathioprine therapy, he was readmitted due to bouts of repeated deep neck swelling, laryngeal edema, and fever. Symptoms were alleviated with antibiotics and fluid therapy, but no causative organism was identified. After his fever resolved, he developed slow progressive azotemia, proteinuria, and microscopic hematuria. An examination revealed swelling of the proximal interphalangeal joints of both hands with swan neck and flexion deformities (Figure 2). His laboratory tests showed his hemoglobin level to be 8.8g/dL, white blood cell count 4340/μL (neutrophils 71%), platelet count 56×103/μL, total protein level 6.0g/dL, albumin level 3.1g/dL, BUN level 22mg/dL, creatinine level 1.99mg/dL, and C-reactive protein level 2.875mg/L. His C3 level was 9.0mg/dL (normal range 90 to 180mg/dL), C4 level was <1.5mg/dL (normal range 10 to 40mg/dL), CH50 level was <2.0U/mL (normal range 23 to 46U/mL), and C1q level was 5.02mg/dL (normal range 11.8 to 23.8mg/dL). A urine analysis showed protein 1+, red blood cell count 1 to 3/high-power field (HPF), and urine protein-creatinine ratio of 1470.6mg/g. The 24-hour urine protein level was 1.6g/day. A percutaneous renal biopsy was performed; the specimen for light microscopy contained 12 glomeruli and 2 arteries up to interlobular size. The glomeruli were enlarged. The mesangium was focally expanded due to an increase of the matrix without hypercellularity and the glomerular capillary walls were mildly thickened. There were patchy areas of interstitial inflammation with mostly mononuclear cells and fibrosis. The tubules showed degenerative changes of the epithelial cells with proteinaceous casts and atrophy and the blood vessels showed arteriolosclerosis. Immunofluorescent staining revealed granular deposits of IgG(++), IgM(+), C3(+/-), C1q(+/-), and C4d(+++) along the capillary loop. An electron microscopy revealed some irregular electron dense deposits in the subendothelial and paramesangial space. His renal biopsy results were compatible with membranoproliferative glomerulonephritis type I (MPGN type I) (Figure 3A,B). He was diagnosed with HUVS with cardiac valvulopathy, Jaccoud’s arthropathy, and MPGN type I. Three daily doses of intravenous methylprednisolone (500mg each) were administered, followed by oral prednisone (1mg/kg/day) and cyclophosphamide (0.8mg/kg/day).

After six months of cyclophosphamide treatment, his treatment was maintained with prednisolone (15mg/day), hydroxychloroquine (400mg/day), and mycophenolate mofetil (MMF, 1.5g/day). His serum creatinine was 1.56mg/dL, his urine analysis showed protein 1+, his red blood cell count was 1 to 3/HPF, and he had a urine protein-creatinine ratio of 291.7mg/g. He remained markedly hypocomplementemic, and continued with slowly progressive severe arthropathy of the hands, despite improved nephropathy.

## Discussion

HUVS is a rare type of vasculitis first described in 1973 by McDuffie *et al*. [6] characterized clinically by recurrent urticaria with a variety of systemic manifestations. In 1982, Schwartz *et al.* established the accepted diagnostic criteria [7]. Two major criteria (recurrent urticaria lasting longer than six months and hypocomplementemia) and at least two minor criteria (venulitis on skin biopsy, arthralgias or arthritis, glomerulonephritis, ocular inflammation, abdominal pain, and positive C1q antibodies) are required for the diagnosis of HUVS. In some patients, HUVS may be secondary to underlying systemic lupus erythematosus (SLE), Sjogren syndrome, or neoplasia [8,9].

Our patient matched the criteria for HUVS on the basis of the clinical findings of recurrent urticaria, arthritis, and abdominal pain, as well as laboratory findings of marked continuous hypocomplementemia and pathological findings of cutaneous vasculitis and glomerulonephritis, in the absence of any other secondary cause.

HUVS primarily occurs in young women, with onset between 23 and 66 years of age with a peak incidence in the fourth decade of life [1,10]. Obstructive lung disease, angioedema, ocular inflammation, and glomerulonephritis are common manifestations in HUVS [11]. Lung disease is the principal cause of morbidity and mortality. Wisnieski *et al.*[1] described 18 patients with HUVS; 50% of patients had renal involvement that varied from minimal proteinuria to nephrotic syndrome. Six renal biopsies were performed; three cases showed mesangial proliferative glomerulonephritis, two showed MPGN, and one showed membranous nephropathy [1].

There is no consensus on the appropriate treatment of HUVS. Corticosteroid therapy is reportedly effective in many cases of HUVS [11,12], though several cases in adults have progressed to end-stage renal disease [13]. Our patient developed MPGN three years after initial presentation, despite corticosteroid and azathioprine therapy. He continued with progressive worsening of kidney function. He subsequently showed a partial response to a combination of corticosteroids, cyclophosphamide, and MMF.

Some patients with HUVS have had concomitant cardiac valvulopathy and Jaccoud’s arthropathy. In 1993, Palazzo *et al.* described three Caucasian patients with Jaccoud’s arthropathy, HUVS, and cardiac valvulopathy [2]. Since 2000, three other patients (an African-American male, a white female, and a Japanese female) have shown this rare combination of symptoms [3-5]. All six of these patients showed no detectable antibodies to C1q and had no kidney involvement. Our patient had MPGN along with arthropathy, valvulopathy, and HUVS, however, he was not tested for antibodies to C1q.

In 2002, Houser *et al.* reported the histological findings of the cardiac valves in these patients [14]. His histopathology tests revealed vasculitis with fibrinoid necrosis and granulation tissue with numerous inflammatory cells. One patient showed C1q staining in the valve, and another patient showed prominent deposition of IgG, IgM, and small amounts of IgA on the valve surface with immunostaining [14].

In the present case, our patient showed extensive immunoglobulin and complement deposition in the glomerular capillary loop. The presence of complement and immunoglobulin in the valvulopathy and glomerulonephritis specimens may be more consistent with damage induced by an immune complex mechanism rather than by an antibody, as in HUVS.

Our patient was the seventh case of the concurrence of HUVS, Jaccoud’s arthropathy, and cardiac valvulopathy. He also developed azotemia associated with membranoproliferative glomerulonephritis, unlike other cases. He was treated with a combination of corticosteroids, cyclophosphamide, and MMF, which stabilized his nephropathy. But his cutaneous vasculitis, cardiac valvulopathy, and arthropathy remained unchanged.

## Conclusions

We describe, to the best of our knowledge, the first case of glomerulonephritis along with arthropathy, cardiac valvulopathy, and HUVS. A combination of corticosteroids, cyclophosphamide, and MMF appear to be a safe and effective treatment of nephropathy, however, are less effective for cutaneous vasculitis, cardiac valvulopathy, and arthropathy.

## Consent

Written informed consent was obtained from the patient for publication of this case report and any accompanying images. A copy of the written consent is available for review by the Editor-in-Chief of this journal.

## Abbreviations

ANA: Antinuclear antibodies; anti-CCP: Anti-cyclic citrullinated peptide antibody; ANCA: Antineutrophil cytoplasmic antibody; BUN: Blood urea nitrogen; CT: Computed tomography; ESR: Erythrocyte sedimentation rate; HUVS: Hypocomplementemic urticarial vasculitis syndrome; MMF: Mycophenolate mofetil; MPGN: Membranoproliferative glomerulonephritis; SLE: Systemic lupus erythematosus.

## Competing interests

The authors declare that they have no competing interests.

## Authors’ contributions

HCC was in charge of the patient’s treatment and care and was principal writer of the manuscript. CP and SWC were involved in the overall care of the patient. JP and JSL were involved in the investigation of data. MK assisted in manuscript preparation. All authors read and approved the final manuscript.
